# Supplementary material for: Polygenic risk score-based phenome-wide association study identifies novel associations for Tourette syndrome
Source: Transl Psychiatry. 2023 Feb 23;13:69. doi: 10.1038/s41398-023-02341-5 (PMC9950421; doi:10.1038/s41398-023-02341-5)
Supplement: Supplementary file 1 — Supplementary document [file 41398_2023_2341_MOESM1_ESM.pdf]

Supplement to “Polygenic Risk score - based phenome wide association studies identify novel associations for Tourette’s Syndrome”

Jain et al.

List of tables

1	GWAS studies and Number of SNPs used for PRS calculation	2
2	Phenotype sub-categories	3
3a	TS PheWAS significant associations – Biochemical Measures	4
3b	TS PheWAS significant associations – Cognition and Mental Health	4
3c	TS PheWAS significant associations – Disease Diagnosis	5
3d	TS PheWAS significant associations – Health and Medical History	5
3e	TS PheWAS significant associations – Sociodemographics	6
4	Significant associations – PheWAS female	7
5	Significant associations – PheWAS male	8

List of figures

1	Significant associations with TS PRS after FDR correction	9

References 10

Supplementary table 1: GWAS studies used for PRS Calculations

Disorder	Cases	Controls	N SNPs (PRS)	Reference
TS	6,133	13,565	307,955	(1)
ADHD	20,183	35,191	924,303	(2)
ASD	18,381	27,969	958,504	(3)
OCD	2,688	7,037	964,437	(4)

Supplementary table 2: List of Sub-categories of phenotypes

Category	Sub-Category	Number of Phenotypes
Biochemical Measures	Antigens	56
Biochemical Measures	Blood assays	96
Biochemical Measures	Serostatus	13
Biochemical Measures	Urine assays	4
Cognition and Mental Health	Addictions	10
Cognition and Mental Health	Anxiety	31
Cognition and Mental Health	Depression	34
Cognition and Mental Health	Fluid intelligence and trail making	10
Cognition and Mental Health	Happiness and subjective well-being	3
Cognition and Mental Health	Mania	12
Cognition and Mental Health	Mental distress	18
Cognition and Mental Health	Pairs matching	6
Cognition and Mental Health	Psychosocial factors	49
Cognition and Mental Health	Self-harm behaviours	19
Cognition and Mental Health	Symbol digit substitution	6
Cognition and Mental Health	Traumatic events	15
Cognition and Mental Health	Unusual and psychotic experiences	9
Disease Diagnosis	circulatory system	136
Disease Diagnosis	congenital anomalies	48
Disease Diagnosis	dermatologic	79
Disease Diagnosis	digestive	135
Disease Diagnosis	endocrine/metabolic	116
Disease Diagnosis	genitourinary	133
Disease Diagnosis	hematopoietic	46
Disease Diagnosis	infectious diseases	50
Disease Diagnosis	injuries & poisonings	90
Disease Diagnosis	mental disorders	64
Disease Diagnosis	musculoskeletal	105
Disease Diagnosis	neoplasms	123
Disease Diagnosis	neurological	73
Disease Diagnosis	pregnancy complications	30
Disease Diagnosis	respiratory	70
Disease Diagnosis	sense organs	106
Disease Diagnosis	symptoms	25
Health and Medical History	Digestive health	33
Health and Medical History	Family history	93
Health and Medical History	Health and medical history	102
Health and Medical History	supplements and medication	27
Health and Medical History	Pain Related Phenotypes	21
Sociodemographics	Early life factors	9
Sociodemographics	Greenspace and coastal proximity	9
Sociodemographics	Home and work locations	3
Sociodemographics	Indices of Multiple Deprivation	24
Sociodemographics	Characteristics and reception	4
Sociodemographics	Residential air and noise pollution	20
Sociodemographics	Sex-specific factors	31
Sociodemographics	Sociodemographics	52

Supplementary table 3a: Phenotypes significantly associated with TS PRS – Biochemical Measures category

**Phenotype**	**beta**	**se**	**pvalue**
Glycated hemoglobin (HbA1c)	0.007990481	0.001717972	3.30E-06

Supplementary table 3b: Phenotypes significantly associated with TS PRS – Cognition and Mental Health category

**Phenotype**	**beta**	**se**	**pvalue**
Seen doctor (GP) for nerves, anxiety, tension or depression	0.031276575	0.003761667	9.19E-17
Neuroticism score	0.026540103	0.003360914	2.87E-15
Tense / 'highly strung'	0.03453692	0.004766234	4.28E-13
Worrier / anxious feelings	0.026067196	0.003641953	8.21E-13
Sensitivity / hurt feelings	0.02231224	0.003657122	1.05E-09
Frequency of tiredness / lethargy in last 2 weeks	0.018950542	0.003388182	2.23E-08
Ever unenthusiastic/disinterested for a whole week	0.035871683	0.006477429	3.05E-08
Mood swings	0.019211779	0.003604395	9.81E-08
Fed-up feelings	0.019126261	0.003648398	1.58E-07
Worry too long after embarrassment	0.018965852	0.003628507	1.72E-07
Irritability	0.021010278	0.004033484	1.90E-07
Frequency of tenseness / restlessness in last 2 weeks	0.021037343	0.004042269	1.95E-07
Seen a psychiatrist for nerves, anxiety, tension, or depression	0.028632456	0.005571249	2.75E-07
Nervous feelings	0.021483792	0.004207695	3.29E-07
Ever depressed for a whole week	0.031597142	0.006198293	3.43E-07
Ever suffered mental distress preventing usual activities	0.0335722	0.006653632	4.51E-07
Ever had prolonged loss of interest in normal activities	0.031359828	0.006387983	9.13E-07
Physically abused by family as a child	0.038067792	0.007926903	1.57E-06
Miserableness	0.016949836	0.003637477	3.16E-06
Substances taken for anxiety: Medication prescribed to you	0.04075991	0.009197465	9.33E-06
Ever sought or received professional help for mental distress	0.028184137	0.00640976	1.10E-05

Supplementary table 3c: Phenotypes significantly associated with TS PRS – Disease Diagnosis category

**Phenotype**	**beta**	**se**	**pvalue**
Depressive episode	0.051782103	0.007752179	2.38E-11
Back pain	0.04029887	0.00858208	2.65E-06
Anxiety disorder	0.037252578	0.0093597	6.89E-06
Other diseases of respiratory system, NEC	0.040522754	0.009037249	7.33E-06
Peripheral enthesopathies and allied syndromes	0.052264889	0.011754039	8.72E-06
Abdominal pain	0.023425516	0.005279084	9.10E-06
Palpitations	0.063867017	0.014697361	1.39E-05
Type 2 diabetes	0.028680425	0.006884423	2.10E-05

Supplementary table 3d: Phenotypes significantly associated with TS PRS – Health and Medical History category

**Phenotype**	**beta**	**se**	**pvalue**
Pain type(s) experienced in last month: Back pain	0.026955263	0.004044663	2.65E-11
Pain type(s) experienced in last month: Neck or shoulder pain	0.026363456	0.00420298	3.55E-10
Overall health rating	0.019057118	0.003412573	2.35E-08
Taking other prescription medications	0.019017416	0.003605174	1.33E-07
Medication for pain relief,heartburn: Paracetamol	0.022608598	0.00430526	1.51E-07
Hearing difficulty/problems with background noise	0.018863865	0.003711667	3.73E-07
Pain type(s) experienced in last month: Knee pain	0.021360894	0.004312795	7.31E-07
Long-standing illness, disability, or infirmity	0.018801412	0.003838125	9.65E-07
Shortness of breath walking on level ground	0.049070123	0.010064553	1.08E-06
Degree bothered by back pain in the past 3 months	0.026532799	0.005464276	1.20E-06
Sensitive stomach	0.035381412	0.007307202	1.28E-06
Degree bothered by pain in arms/leg in the past 3 months	0.026394204	0.005462537	1.35E-06
Pain type(s) experienced in last month: Hip pain	0.024873744	0.005601627	8.98E-06
Mouth/teeth dental problems: Mouth ulcers	0.025362618	0.005787062	1.17E-05

Supplementary table 3e: Phenotypes significantly associated with TS PRS – Socio-demographics category

**Phenotypes**	**beta**	**se**	**pvalue**
Qualifications: College or University degree	-0.029863633	0.003824271	5.79E-15
Age completed full time education	-0.029291899	0.003964888	1.50E-13
Education score (England)	0.012072247	0.001861634	8.91E-11
Employment score (England)	0.009867967	0.00182865	6.81E-08
Qualifications: A levels/AS levels or equivalent	-0.021373731	0.003997793	8.99E-08
Age at first live birth	-0.014886433	0.002822011	1.33E-07
Unable to work because of sickness or disability	0.049014332	0.009509013	2.54E-07
Place of birth in UK - north co-ordinate	0.006688758	0.001383233	1.33E-06
Health score (England)	0.00845566	0.00179795	2.57E-06
Home location at assessment - north co-ordinate (rounded)	0.006857597	0.001507999	5.43E-06
Disability living allowance	0.03869344	0.008843724	1.21E-05
Index of Multiple Deprivation (England)	0.008011607	0.00185223	1.52E-05
Home location - north co-ordinate (rounded)	0.006414243	0.001498488	1.87E-05

Supplementary table 4: Phenotypes significantly associated with TS PRS in females

**Phenotypes**	**beta**	**se**	**pvalue**
Sensitivity / hurt feelings	0.024554381	0.005066822	1.26E-06
Ever unenthusiastic/disinterested for a whole week	0.038756164	0.008631537	7.10E-06
Worry too long after embarrassment	0.020998993	0.004907105	1.87E-05
Seen doctor (GP) for nerves, anxiety, tension or depression	0.027269906	0.004873705	2.20E-08
Ever depressed for a whole week	0.039459788	0.008545798	3.87E-06
Worrier / anxious feelings	0.022729625	0.005048433	6.72E-06
Neuroticism score	0.024657801	0.004602478	8.45E-08
Other diseases of respiratory system, NEC	0.061261966	0.013013583	2.50E-06
Depressive episode	0.049273606	0.009746161	4.28E-07
Degree bothered by back pain in the past 3 months	0.035989928	0.007246929	6.84E-07
Degree bothered by dizziness in the last 3 months	0.036256666	0.008350041	1.41E-05
Age at first live birth	-0.01491267	0.002826986	1.33E-07
Age completed full time education	-0.02613301	0.005352668	1.05E-06
Qualifications: College or University degree	-0.03380712	0.005291744	1.68E-10
Education score (England)	0.011996824	0.002537246	2.27E-06

Supplementary table 5: Phenotypes significantly associated with TS PRS in males

Phenotypes	beta	se	pvalue
Seen doctor (GP) for nerves, anxiety, tension, or depression	0.037244529	0.005921185	3.17E-10
Worrier / anxious feelings	0.029714563	0.005260192	1.61E-08
Neuroticism score	0.02871147	0.004918909	5.33E-09
Tense / 'highly strung'	0.046998513	0.007451148	2.83E-10
Seen a psychiatrist for nerves, anxiety, tension, or depression	0.038160614	0.008612896	9.37E-06
Frequency of tiredness / lethargy in last 2 weeks	0.022795959	0.005055506	6.51E-06
Irritability	0.031414525	0.005761787	4.97E-08
Mood swings	0.025237894	0.005333735	2.22E-06
Type 2 diabetes	0.038116973	0.008950332	2.05E-05
Palpitations	0.121221502	0.025119212	1.39E-06
Depressive episode	0.056085476	0.012804602	1.19E-05
Long-standing illness, disability, or infirmity	0.025966399	0.005536675	2.73E-06
Pain type(s) experienced: Back pain	0.033509639	0.005886159	1.25E-08
Pain type(s) experienced: Neck or shoulder pain	0.02983633	0.006318959	2.34E-06
Education score (England)	0.012046066	0.002739844	1.10E-05
Qualifications: A levels/AS levels or equivalent	-0.02673037	0.005882126	5.52E-06
Qualifications: College or University degree	-0.02566017	0.005540527	3.64E-06
Age completed full time education	-0.03319891	0.005903219	1.87E-08

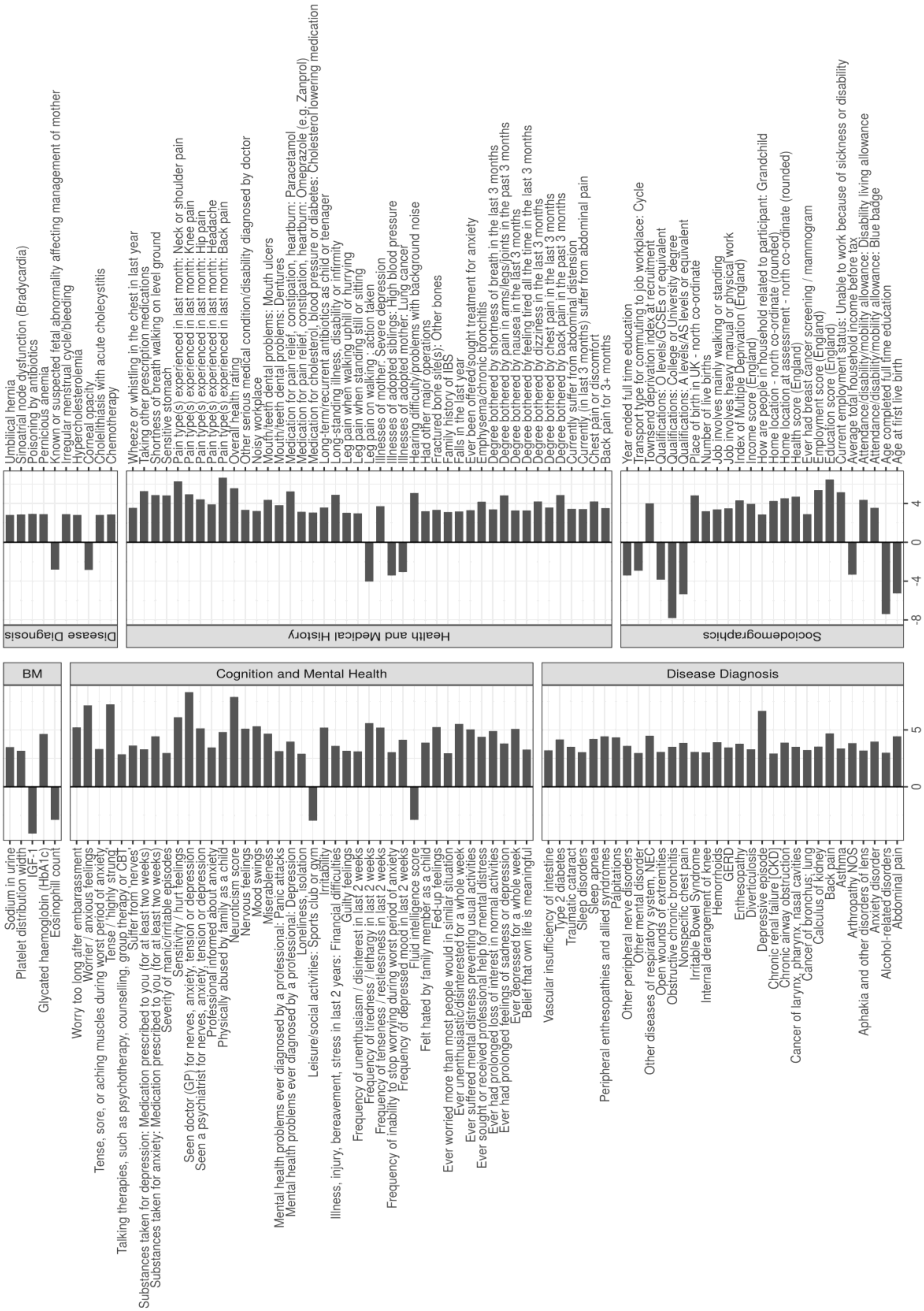

Supplementary Figure 1: Phenotypes Significantly associated with TS PRS after FDR correction arranged by categories. BM: Biochemical measures. Bars Indicate Z-score of association.

References

1. Tsetsos F, Topaloudi A, Jain P, Yang Z, Yu D, Kolovos P, et al. Genome-wide Association Study identifies two novel loci for Gilles de la Tourette Syndrome. medRxiv [Internet]. 2021 Dec 13 [cited 2022 Feb 9];17:2021.12.11.21267560.

2. Demontis D, Walters RK, Martin J, Mattheisen M, Als TD, Agerbo E, et al. Discovery of the first genome-wide significant risk loci for attention deficit/hyperactivity disorder. Nat Genet. 2019;

3. Grove J, Ripke S, Als TD, Mattheisen M, Walters RK, Won H, et al. Identification of common genetic risk variants for autism spectrum disorder. Nat Genet. 2019;51(3):431–44.

4. Arnold PD, Askland KD, Barlassina C, Bellodi L, Bienvenu OJ, Black D, et al. Revealing the complex genetic architecture of obsessive-compulsive disorder using meta-analysis. Mol Psychiatry. 2018;
